# Surgical Resection of a Pseudoaneurysm of the First Dorsal Metatarsal Artery after Unsuccessful Embolization: A Case Report and Literature Review

**DOI:** 10.70352/scrj.cr.24-0020

**Published:** 2025-01-31

**Authors:** Hiroto Yasumura, Kenichi Arata, Goichi Yotsumoto, Hideyuki Satozono, Koichiro Shimoishi, Yoshihiro Fukumoto, Yuki Ogata, Tomoyuki Matsuba, Yoshiharu Soga

**Affiliations:** 1 Department of Cardiovascular Surgery, Kagoshima City Hospital, Kagoshima, Kagoshima, Japan; 2 Department of Cardiovascular Surgery, Graduate School of Medical and Dental Sciences, Kagoshima University, Kagoshima, Kagoshima, Japan

**Keywords:** peripheral artery aneurysm, pedis artery aneurysm, metatarsal artery aneurysm, embolization, surgery

## Abstract

**INTRODUCTION:**

Aneurysms of peripheral foot arteries are extremely rare. Dorsalis pedis artery aneurysms account for 0.5% of peripheral artery aneurysms of the lower limbs. Here, we present a case of pseudoaneurysm of the first dorsal metatarsal artery of the foot and discuss the therapeutic strategy based on a literature review.

**CASE PRESENTATION:**

A 76-year-old man with no history of foot trauma presented with pain and a pounding mass in the dorsum of the left foot. Echography revealed a 29 × 18 × 20 mm saccular aneurysm with to-and-fro blood flow. Contrast-enhanced computed tomography revealed an aneurysm in the first dorsal metatarsal artery. Angiography of the aneurysm revealed no arterial drainage. Embolization was subsequently performed only for the feeding artery, which was the proximal first dorsal metatarsal artery, using the 2 Target nanocoils (Stryker; Boston, MA, USA), resulting in successful occlusion. However, echography performed a few months after embolization revealed a recurrence of blood flow and enlargement of the coiled aneurysm. Nine months after embolization, the pain in the dorsum of the foot recurred. Therefore, we performed a surgical resection of the dorsal metatarsal artery aneurysm (38 × 26 × 26 mm) under general anesthesia. The first distal dorsal metatarsal artery exhibited pulsatile bleeding, and angiography of the distal dorsal metatarsal artery revealed a patent pedal arch and posterior tibial artery. Therefore, revascularization was not performed. The postoperative course was uneventful. The pathological examination indicated that the mass was a pseudoaneurysm.

**CONCLUSIONS:**

The treatments for peripheral foot artery aneurysms include observation, thrombin injection, ultrasound compression, embolization, surgical excision, and ligation. As the long-term outcomes of embolization for such aneurysms are unknown and cases are limited, surgical excision that is safe and definitive is recommended as the first-line treatment.

## Abbreviations

DPA
: dorsalis pedis artery
PTA
: posterior tibial artery
DMA
: dorsal metatarsal artery
CT
: computed tomography
MPA
: medial plantar artery

## INTRODUCTION

**Fig. 1:**
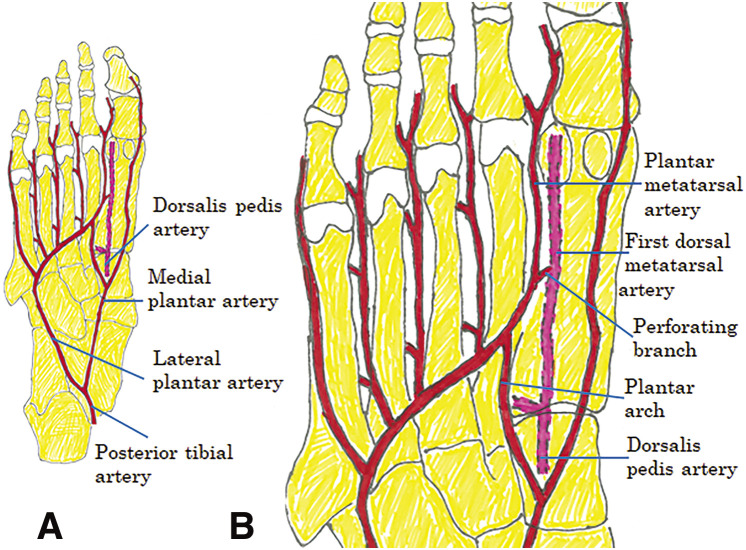
Anatomy of the foot arteries (right foot viewed from the sole). (**A**) The dorsalis pedis and posterior tibial arteries perfuse the foot. (**B**) The dorsal metatarsal artery is located distal to the dorsalis pedis artery. The dorsal and plantar metatarsal arteries are connected by perforating branches.

The dorsalis pedis and posterior tibial arteries perfuse the foot (**Fig. 1A**). The metatarsal arteries are located distal to the dorsalis pedis artery on both the dorsal and plantar sides and are connected by perforating branches (**Fig. 1B**). Aneurysms of peripheral foot arteries are extremely rare. According to Bozio et al.,^1)^ dorsalis pedis artery aneurysms account for 0.5% of peripheral artery aneurysms of the lower limbs. Herein, we present a case of pseudoaneurysm of the first dorsal metatarsal artery and discuss the therapeutic strategy based on a literature review.

## CASE PRESENTATION

**Fig. 2:**
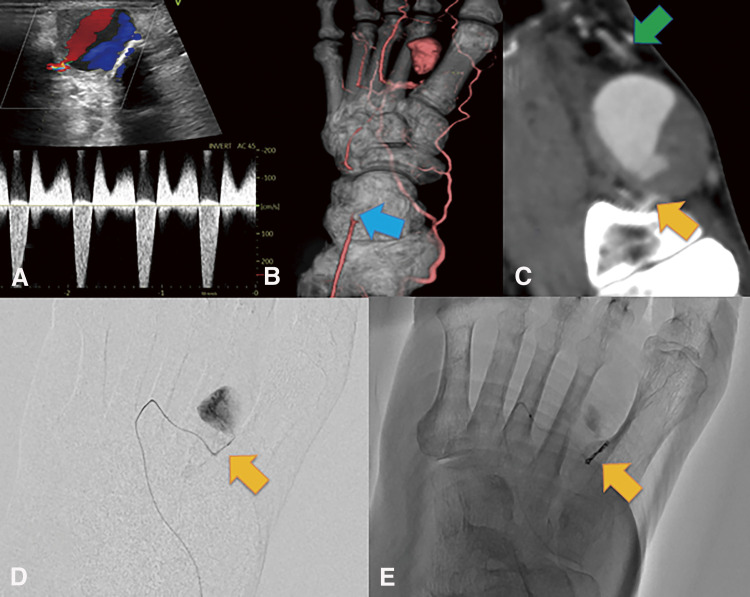
Pre- and intra-embolization findings. (**A**) Echogram indicating a 29 × 18 × 20 mm saccular aneurysm with a to-and-fro blood-flow waveform. (**B**) Contrast-enhanced computed tomography reveals an aneurysm of the first dorsal metatarsal artery and an occluded dorsalis pedis artery (blue arrow). (**C**) The aneurysm is located in the middle segment of the first dorsal metatarsal artery. The orange and green arrows represent the proximal and distal dorsal metatarsal arteries, respectively. (**D**) Digital subtraction angiography of the aneurysm indicates no arterial drainage. The orange arrow indicates the first proximal dorsal metatarsal artery. (**E**) Embolization is performed for the proximal first dorsal metatarsal artery (orange arrow) with Target Nano 2 mm × 3 cm and 1.5 mm × 3 cm (Stryker; Boston, MA, USA).

**Fig. 3:**
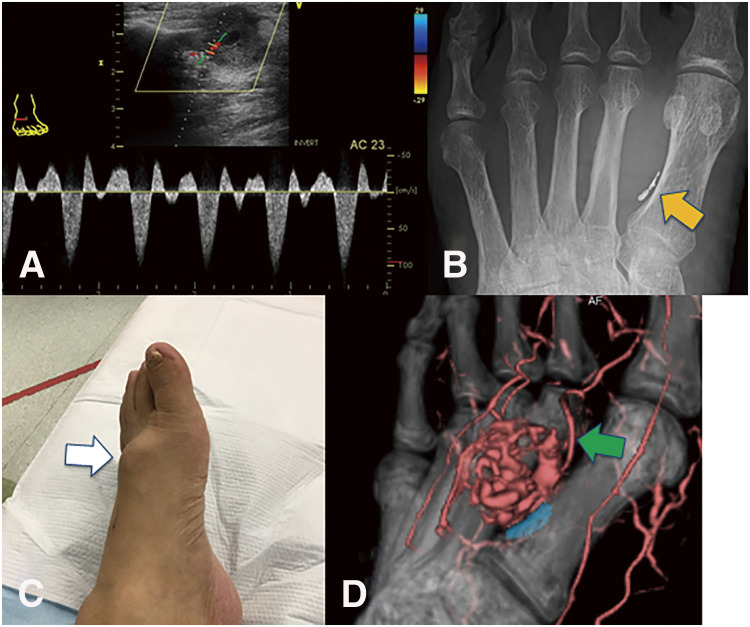
Post-embolization findings. (**A**) Echography performed 3 months after embolization reveals recurrence of the first dorsal metatarsal artery aneurysm with to-and-fro blood flow waveform. (**B**) The radiograph indicates no coil migration (orange arrow). (**C**) Nine months after embolization, the mass on the dorsal foot returned (white arrow), with a recurrence of pain. (**D**) Contrast-enhanced computed tomography reveals that the distal first dorsal metatarsal artery (green arrow) supplies the aneurysm.

**Fig. 4:**
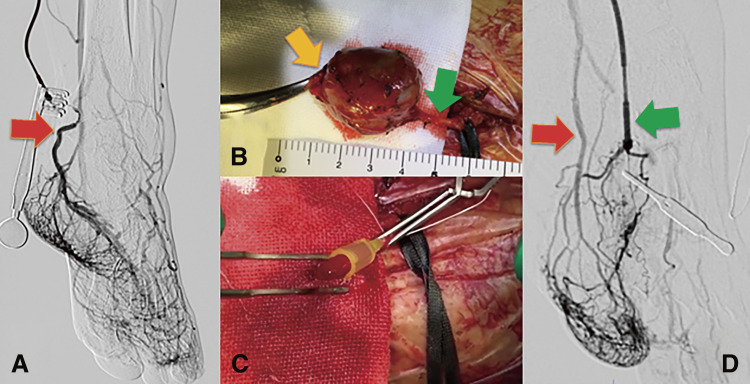
Intraoperative findings. (**A**) Angiography from the posterior tibial artery (red arrow) exhibits no aneurysmal staining. (**B**) The proximal first dorsal metatarsal artery (orange arrow) is ligated, and the previous coils are removed. The distal first dorsal metatarsal artery (green arrow) is cannulated. (**C**) Pulsatile bleeding from the cannulated distal dorsal metatarsal artery is observed. (**D**) Angiography from the distal first dorsal metatarsal artery (green arrow) reveals a patent pedal arch and posterior tibial artery (red arrow).

**Fig. 5:**
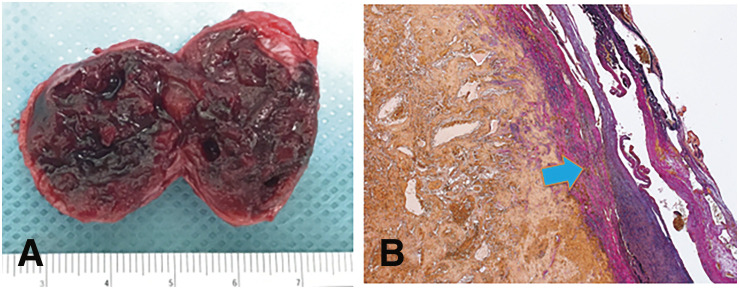
Pathological findings. (**A**) The dorsal metatarsal artery aneurysm is filled with organized thrombus. This image was obtained immediately after removal. (**B**) The wall of the aneurysm consists of fibrous tissue with few smooth muscle cells (blue arrow).

A 76-year-old man with no history of foot trauma presented with a painful mass on the left foot dorsum. A physical examination revealed a pulsatile mass in the first intermetatarsal region. Doppler echography revealed a 29 × 18 × 20 mm saccular aneurysm with a to-and-fro blood flow waveform (**Fig. 2A**). Contrast-enhanced computed tomography (CT) revealed an aneurysm of the first dorsal metatarsal artery and an occluded dorsalis pedis artery (**Fig. 2B**). The aneurysm was located in the middle segment of the first dorsal metatarsal artery (**Fig. 2C**). Angiography of the first dorsal metatarsal artery via medial plantar artery revealed a narrow linear bloodstream into the aneurysm but no drainage vessel (**Fig. 2D**). Therefore, embolization was performed only for the feeding artery that was the proximal first dorsal metatarsal artery, using Target Nano at 2 mm × 3 cm and 1.5 mm × 3 cm (Stryker; Boston, MA, USA) (**Fig. 2E**). Post-procedural angiography revealed successful coiling, and the hematoma was aspirated percutaneously. Pain and pulsation of the aneurysm disappeared. Echography revealed no blood flow in the aneurysm. Three months after embolization, repeat echography revealed the recurrence of the first dorsal metatarsal artery aneurysm with to-and-fro blood flow (**Fig. 3A**). Radiography revealed no coil migration (**Fig. 3B**). Nine months after embolization, the pain relapsed in the foot mass (**Fig. 3C**), and repeat echography revealed an enlarged aneurysm (38 × 26 × 26 mm) with increased blood flow. A contrast-enhanced CT revealed a new feeding artery distal to the first dorsal metatarsal artery (**Fig. 3D**). Therefore, we decided to perform a surgical resection. Angiography of the posterior tibial artery performed under general anesthesia revealed no aneurysmal staining (**Fig. 4A**). A linear incision was made over the mass. The proximal first dorsal metatarsal artery was ligated, and the previous coils were removed (**Fig. 4B**). The distal first dorsal metatarsal artery was ligated, and the aneurysm was excised. The distal dorsal metatarsal artery was cannulated, and pulsatile bleeding was observed (**Fig. 4C**). Angiography of the distal dorsal metatarsal artery revealed a patent pedal arch and posterior tibial artery (**Fig. 4D**). Therefore, revascularization was not required. The postoperative course was uneventful. Pathological examination revealed that the aneurysm was filled with an organized thrombus (**Fig. 5A**), and the wall consisted of fibrous tissue with rare smooth muscular cells (**Fig. 5B**), consistent with a pseudoaneurysm.

## DISCUSSION

Cuff^2)^ first reported a dorsalis pedis artery aneurysm in 1907. Upon searching PubMed using the term “dorsalis pedis artery aneurysm,” approximately 30 case reports detailing these aneurysms can be found. Dorsal metatarsal arterial aneurysms are rare. Only 3 case reports of dorsal metatarsal artery aneurysms were found on PubMed and Google Scholar using the terms “metatarsal artery aneurysm” and “metatarsal pseudoaneurysm” (**Table 1**). One case^3)^ was a true aneurysm, and the other 2 cases^4,5)^ were pseudoaneurysms associated with orthopedic procedures.

**Table 1:** Characteristics of patients with dorsal metatarsal artery aneurysm based on published studies from PubMed and Google Scholar (Accessed on August 18, 2024)

Author	Published year	Age/sex	Aneurysmal location	Trauma or event	Diameter(mm)	Therapy	Pathology
Ysa et al.^3)^	2007	57/F	First DMA	None	12 × 10	Resection and revascularization with microscopic direct end-to-end anastomosis	True aneurysm
Lee et al.^4)^	2014	55/F	First DMA	Correction of hallux valgus	13 × 16 × 12	Resection without revascularization	Pseudoaneurysm
Kinter and Hodgkins^5)^	2019	67/F	Second DMA	Removal of hardware from the lateral naviculocuneiform joint	25 × 17	Resection without revascularization	Pseudoaneurysm
Present case	2024	76/M	First DMA	None	29 × 18 × 20	Embolization →Resection without revascularization	Pseudoaneurysm

DMA, dorsal metatarsal artery

Pseudoaneurysms of the foot are typically caused by trauma;^6)^ however, no history of trauma was observed in our patient. Sonntag et al.^7)^ reported bilateral dorsalis pedis artery true aneurysms due to tight “sandal strap’’ trauma. Our patient was retrospectively confirmed to be wearing tight sandals with a frontal sandal strap between the toes, and the location of the first dorsal metatarsal artery aneurysm was where the strap compressed the first dorsal metatarsal artery from the anterior and superior sides.

Tempest and Wilson^8)^ reported acute forefoot ischemia that followed a dorsalis pedis artery aneurysm and required transmetatarsal amputation, decreasing the patient’s quality of life. Therefore, similar to other aneurysms, treatment of peripheral foot artery aneurysms is recommended. Embolization of dorsalis pedis artery and dorsal metatarsal artery aneurysms has not been reported. In the present case, successful embolization of the feeding artery was performed. However, either the distal dorsal metatarsal artery or a small drainage artery that was not detected preoperatively was considered to have become the new feeding artery. Angiogenesis may also have been involved. Angiography performed during embolization indicated that the proximal first dorsal metatarsal artery was the only feeding artery and did not reveal a drainage artery that would otherwise have been embolized. This is considered a limitation of angiography for pseudoaneurysms. Successful embolization of feeding arteries would have resulted in a painful shrinking of the aneurysm, as successful endovascular aortic repair can cause abdominal artery aneurysms to shrink. Therefore, embolization of the feeding arteries of the pseudoaneurysm is an acceptable treatment method; however, due to the high cost of coils and the possibility of recurrence, we recommend that dorsalis pedis artery and dorsal metatarsal artery aneurysms be treated primarily with surgical resection that is safer and more effective than is coil embolization.

According to several reports,^9,10)^ revascularization should be performed in the following instances: (1) when the pedal arch is not well indicated; (2) in children and adolescents; and (3) in patients with risks of worsening arteriosclerosis and diabetes mellitus. In the present case, as the dorsalis pedis artery was occluded, revascularization of the first dorsal metatarsal artery was considered preoperatively. However, intraoperative angiography of the distal first dorsal metatarsal artery revealed a patent pedal arch and posterior tibial artery, eliminating the need for revascularization. This highlights the importance of intraoperative angiography, even in the context of peripheral artery surgery. Ysa et al.^3)^ successfully reconstructed the dorsal metatarsal artery using a microscopic end-to-end anastomosis.

## CONCLUSION

The treatments for peripheral foot artery aneurysms include observation, thrombin injection, ultrasound compression, embolization, surgical excision, and ligation. The long-term outcomes of embolization for such aneurysms are unknown, and cases are limited. By contrast, open surgery is safe and definitive. Therefore, surgical excision is recommended as the first-line treatment.

## ACKNOWLEDGMENTS

We would like to thank Editage (www.editage.jp) for editing the English language.

## DECLARATIONS

### Funding

None.

### Authors’ contributions

HY wrote the initial draft of the manuscript.

KA, GY, and YS wrote the manuscript.

HY, HS, and KA performed the surgery, and HY and KA followed up with the patient.

All authors participated in the treatment of the patients.

All authors have read and approved the final version of the manuscript.

### Availability of data and materials

The dataset supporting the conclusions of this article is included within the article.

### Ethics approval and consent to participate

Not applicable.

### Consent for publication

Consent for the publication of this manuscript was obtained from the patient.

### Competing interests

The authors declare that they have no competing interests.
